# Should Clinically Assisted Hydration and Nutrition Ever Be Withdrawn for a Neonate with a Chronic Non-Progressive Neurological Condition? A Case Study

**DOI:** 10.3390/children12030287

**Published:** 2025-02-26

**Authors:** Zhi-Lin Kang, Keson Tay, Poh-Heng Chong

**Affiliations:** HCA Hospice Limited, Singapore 328127, Singapore; kesont@hcahospicecare.org.sg (K.T.); pohhengc@hcahospicecare.org.sg (P.-H.C.)

**Keywords:** withdrawal of hydration and nutrition, neonatal, chronic non-progressive neurological condition

## Abstract

Background: For infants, withholding or withdrawal of feeding is ethically permissible when the child is imminently dying or chronically and irreversibly comatose. It can also be appropriate in cases of medical futility with a low chance of survival. However, there is much contention in situations where the medical prognosis is uncertain. Case presentation: Annie is a 6-week-old neonate with antenatally acquired cystic encephalomalacia, a chronic non-progressive neurological condition. Her future neurological outcome is uncertain. She is putting on weight in the NICU with stable cardiorespiratory status on room air and tolerates full nasogastric tube feeding but requires frequent oropharyngeal suctioning. Her parents ask to stop tube feeding and allow Annie to die. They deem she has a poor quality of life and is experiencing tremendous suffering. Discussion: Parents’ perceptions of “best interest” and “physical suffering” are explored, alongside those of the healthcare team. Concomitant issues like feeding withdrawal and moral distress are examined in context—that of a newborn where developmental outcomes and disease trajectory are unclear. Conceptual frameworks, empirical evidence and consensus-based ethics guidelines informed a rich and multi-dimensional exposition of a difficult and value-laden decision. Conclusions: While instinctively legitimate, enteral feeding in an infant, in this case with severe neurological impairment, is ultimately still a medical intervention. In contrast to prevailing conventions within adult medicine, the careful and nuanced consideration of benefits and burdens from different stakeholders’ perspectives is critical before any deliberate withdrawal to allow natural death.

## 1. Background

The topic of clinically assisted feeding among dependents is hotly debated, with healthcare professionals holding polarizing views [1]. The provision of hydration and nutrition is seen as a fundamental human right. Strong emotions have lurked behind arguments for its imposition, much more than decisions around mechanical ventilatory support or cardiopulmonary resuscitation (CPR) [2]. A survey by the Pediatric Section of the Society for Critical Care Medicine found that 98% of physicians were apt to withhold CPR, 86% to withdraw ventilators, and only 42% to withdraw tube feeding [3].

The imperative to protect medically threatened infants (with aggressive support) is even more compelling, as they do not yet have the capacity to care for themselves or make their needs known. Nasogastric tube insertion and assisted enteral feeding, for instance, could be regarded as standard of care in the neonatal intensive care unit (NICU). Expectedly, most medical practitioners in this setting find stopping mechanical ventilation^1^ easier than withdrawing feeds. Part of the rationale could be the quick demise of an infant following the cessation of a mechanical ventilator, whereas when feeds are withdrawn, death might take days to short weeks. Discomfort and possibly regrets among caregivers can arise in the interim. Nonetheless, medically provided fluids or nutrition do represent medical interventions, and like all other procedures, careful deliberation of their burdens and benefits is warranted [4].

In the setting of pediatrics in general and neonatology in particular, a delicate clinical challenge can become even more complex—a medically vulnerable infant that is not yet self-determining lies both in the hands of distressed biological parents and professional domain experts. And when a difficult medical decision needs to be made, with life-changing consequences no less, a complex situation naturally emerges.

### Aim

This case report explores moral dilemmas faced by medical professionals in the NICU as one family requests to withdraw artificial hydration and nutrition for a neonate with a chronic non-progressive neurological condition. It describes varying perceptions of suffering and examines how moral uncertainty can impact medical decision-making. The four ethical principles of autonomy, beneficence, non-maleficence and justice will be woven into this discussion, alongside addressing the idea of what suffering means in cases of uncertainty. Implications for clinical practice will be shared at the end.

## 2. Case Presentation

Annie was a 6-week-old neonate, twin 1 of an MCDA (monochorionic diamniotic) pregnancy who was born prematurely at 33 weeks and 2 days old. This was the mother’s first pregnancy, and the antenatal history was unremarkable. The mother was healthy throughout the pregnancy, and she went into spontaneous preterm labor.

Annie was born via emergency cesarean section at 1925 g with Apgar scores of 6 and 9 at 1 and 5 min of life, respectively. She started to develop mild respiratory distress, had recurrent jitteriness on day 1 of life and was transferred to the NICU for continuous positive airway pressure (CPAP). Further investigations were performed, and the MRI brain showed a cystic appearance (left more than right) in the bilateral high parietal subcortical and deep white matter, as well as bilateral precentral gyri white matter with preservation of the cortical grey matter. An electroencephalogram (EEG) showed no seizures. These findings were consistent with leukomalacia due to in utero insults. No acute infarct or intracranial bleeding was evident.

Annie remained in the NICU, requiring intermittent CPAP support with issues of severe oropharyngeal dysphagia, gastroesophageal reflux with feed intolerance and recurrent aspiration. At six weeks, she was stable in room air without respiratory support, on full nasogastric tube feeding but required frequent oropharyngeal suctioning. She had had no seizures and neurologically appeared to be improving over time, but stayed microcephalic. Bedside examination revealed a weak gag and suck response, but she was able to display some visual and auditory awareness. Her neurologist opined that the severity of cerebral palsy was hard to predict, though her cognition was anticipated to be less affected. There were good prognostic factors, as lesions were subcortical, mostly unilateral, and without deep grey matter involvement. Right-sided hemiplegia with delayed motor milestones, as well as speech and swallowing dysfunction were predicted, but more time was needed to fully assess her outcome. A suggested timepoint to more accurately prognosticate her level of disability and severity of cerebral palsy was at 2 years of age. This was because early intervention has been shown to modify and reduce the morbidity of sequelae. Hence, outcomes remained uncertain.

In terms of her social setup, Annie’s dad was a physician, and her mum had stopped working during the pregnancy. Annie’s twin sibling was well and had been discharged home. Extended families from both sides were supportive of the nuclear family, and they were financially stable.

During numerous family discussions, her dad repeatedly brought up their intention to withdraw assisted feeding, as they felt that Annie’s future of likely cerebral palsy was not good quality of life and was prolonging suffering for her. They felt that if Annie was unable to become an independent person in the future, with no need for any support, it was considered a difficult life of suffering. This was compounded by the fact that she had a neurologically normal twin sister, so parents were concerned that Annie might feel even more unjust. The parents believed the feeding tube was a medical device; hence, it was an artificial intervention that brought more harm than benefit, with a high risk of aspiration given her underlying oropharyngeal dysphagia. They highlighted repeatedly that the “window of opportunity” to withdraw feeds was shortening. As the patient grew older, they could lose the opportunity. The father had also enquired about the possibility of stopping suctioning at the time.

There was difference in opinion between the medical team and the parents’ perspectives of what constituted “suffering” and what was considered appropriate “compassionate care”. The parents felt that the future of cerebral palsy (the best-case scenario given was right-sided hemiplegia with intact cognition, while the worst case was severe quadriplegic cerebral palsy with the need for an enteral feeding tube) was both physical and emotional suffering in and of itself and constituted sufficient reasons to withdraw feeds. They also saw the nasogastric tube as a medical device, with risks outweighing benefits, such as the risk of aspiration or tube dislodgement. The medical team were at odds with the parents regarding their opinion. They felt that Annie deserved a chance at survival, as she could have good neurological outcomes with good nutrition and neuro-rehabilitation. Although enteral feeding did constitute a medical intervention, the medical team felt that it was a basic right that all infants should have, and Annie was nowhere near a terminal condition or in a permanent vegetative state to warrant withdrawal. This led to an impasse between the parents and the healthcare team. An ethics committee consultation was offered, but the parents declined, as they felt that the waiting time was too long.

## 3. Discussion

### 3.1. Idea of ‘Suffering’

Suffering is an emotive word that often conjures up images of a child in pain or in distress. Assessing and validating suffering in infants and nonverbal children poses significant challenges due to their inability to articulate effectively.

A qualitative content analysis of pediatric bioethics and clinical literature over 10 years found 651 occurrences of the term “suffering” across 121 articles [5]. The analysis found that the use of “suffering” was ambiguous, and 52% were used to influence decision-making in some way. They also found that claims of a patient suffering were three times more likely to support a life-ending decision versus a life-extending decision (32% vs. 10%).

The idea of suffering in children and neonates, who are universally acknowledged as a vulnerable population dependent on others to advocate for their best interests and well-being, places further emphasis on the weight of the decision between life-ending and life-extending measures. When we are advocating for these vulnerable little lives, medical teams often seek concrete and objective measures of suffering, such as clear and indisputable evidence of medical futility or organ failure, only after which difficult decisions might be made with confidence.

**Figure 1:**
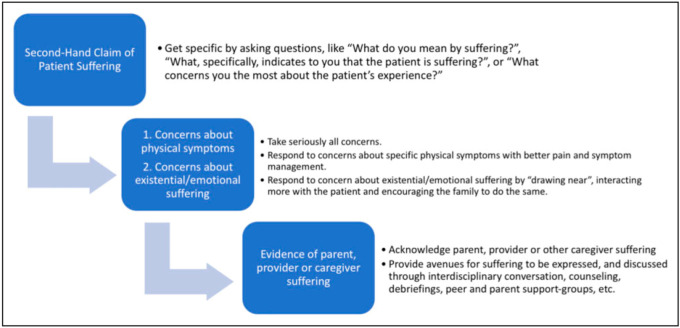
Recommendations for responding to claims of patient suffering.

In her article, Erica Salter argues that “like “futility”, claims of patient “suffering” have been used (perhaps sometimes consciously, but most often unconsciously) to smuggle value judgments about quality of life into decision-making” [6]. She argues that using the blanket term “suffering” may conceal the speaker’s own concerns about the patients. This illustrates the importance of good communication in uncovering what “suffering” means to the caregiver and the healthcare team. She gives recommendations for responding to claims of patient suffering (see Figure 1) [6] (p. 9).

For this case, perspectives and definitions of suffering from different stakeholders conflict and, consequently, complicate the decision-making process. Studies have shown that parents, have differing views and definitions of what constitutes suffering [7], with some parents identifying physical pain as suffering, while some define suffering as having little to no hope for improvement. A more objective theory of pediatric suffering is required to account for the complex relationship between the parent and the child [8]. The idea of future suffering should also be explored specifically so that a good balance of risks and benefits of the current treatment (i.e., enteral feeding and suctioning) can be achieved, which will aid in decision-making. Annie’s parents, as advocates for Annie’s autonomy, had different perspectives from the medical team regarding Annie’s suffering, leading to the impasse in clinical decision-making described above.

### 3.2. Moral Distress of Medical Team

It is the primary goal of the medical team to alleviate suffering in all forms in which it is perceived, and the difficulty in doing so in this case led to substantial moral distress.

According to Jameton’s highly influential definition, moral distress occurs when a nurse knows the morally correct action to take but is constrained in some way from taking this action [9]. These constraints are external and could be due to institutional constraints or physician’s decisions. However, there have been criticisms of this definition, as it is too narrow. It fails to address situations where there is moral uncertainty, and it does not include other healthcare professionals, such as physicians or social workers [10].

Moral uncertainty can arise from conflict, dilemma or uncertainty due to differing value systems and should be acknowledged as causing distress to healthcare professionals. Studies have shown that moral distress leads to burnout and higher turnover intent [11,12]. It is crucial to create well-functioning moral communities within any healthcare institution so that everyone is included and their voices heard [13]. Efforts should be made to reach out to the medical team to understand their concerns while acknowledging that the distress they feel is real and legitimate.

In this case, a major source of constraint faced by the medical staff was uncertainty. The medical team was in distress, as they felt that Annie still had a reasonable chance of doing well and that enteral feeding was a basic care need. The ethics team can help to address their distress by refining the question posed so that they can distinguish between the differences in value judgment of the caregivers and the medical team. It is also important to highlight to the medical team that although withdrawing feeds is morally permissible, it is not morally required, and efforts should be made to engage the medical team in a non-judgmental manner such that they are free to act or not act in a manner that is contradictory to their values.

### 3.3. So, What Now? Withholding Nutrition and Hydration

When deciding on the withdrawal of nutrition and hydration in infants, we need to consider both medical and human value facts [14]. Medical facts include considering the underlying diagnosis, response to previously given treatments, likely response to appropriate treatments or interventions not yet offered and the ultimate prognosis for the infant’s condition. Annie was showing signs of dysphagia with the pooling of secretions and had had one prior episode of aspiration pneumonia. She was at high risk of having another episode of aspiration pneumonia, even with tube feeding. Human value facts consist of what the parents valued and expected or desired for their child, as well as values upheld by the healthcare team. Certainly, parents—being the main caregivers—were considered the decision-makers for their child, as they would be the ones looking after the child once discharged from the hospital. Parents deemed that the possibility of severe spastic cerebral palsy was a life not worth living, and having a normal twin could make this reality worse. Just like we alluded to earlier, more time and effort in communication should be made to find out what parents define as suffering and what extent of disability is acceptable to parents. Ongoing discussion with neonatologists and neurologists, combined with the benefit of time and observation of her clinical progress, could lead to a more accurate prognosis and likelihood of whether Annie might achieve the stated goal. However, this was in conflict with her parents’ wishes, as they perceived that prolonging life with the intention of further observation for an accurate prognosis directly increased Annie’s suffering in the short term and further narrowed the window for withdrawal of life-sustaining therapy. Her parents, therefore, interpreted the medical decision not to withdraw feeds as one that would harm their daughter in the long run.

Based on the latest framework published by the Royal College of Paediatrics and Child Health (RCPCH) in the UK on making decisions to limit treatment in life-limiting and life-threatening conditions in children, there are three sets of circumstances in which treatment limitation should be considered because it is no longer in the child’s best interests to continue [15]. They are as follows: when life is limited in quantity or quality and when there is informed competent refusal of treatment, like older children who may have extensive experience of illness. Regarding the issue of a limited quantity of life, Annie was not facing imminent death, and she also did not have an expected short prognosis; that is, demise inevitable, as in the cases of certain untreatable metabolic conditions.

The area of contention here is the definition of what constitutes a life that is limited in quality. RCPCH breaks down situations when life is limited in quality. Firstly, where burdens of treatments are high so that they outweigh any potential or actual benefits. In this case, enteral tube feeding did not produce so much pain and suffering so as to outweigh its benefits of sustaining life. Certainly, nutrition is needed for growth, metabolism and immunity in preterm infants. Suctioning may be uncomfortable, but the feeling of oral secretions pooling in the infant’s airway may also bring about discomfort. Hence, we argue that the burden of the current treatment is not high. However, as Annie’s parents had implied, the future care needs for a neurologically impaired child are high, which would have a direct impact on the parents’ ability to care for both Annie and her twin sibling. This could potentially exacerbate tensions within the family, given how different the care needs of each child are likely to be.

Secondly, it is appropriate to consider treatment limitation when “the severity and impact of the child’s underlying condition is itself sufficient to produce such pain and distress as to overcome any potential or actual benefits in sustaining life”. Thirdly, the child’s condition is so severe that it is difficult for them to derive benefit from continued life. Collectively, we argue that at 6 weeks old, it is unreasonable to ascertain if the extent of cerebral palsy is severe enough to deter any potential or actual benefits in sustaining life. This is entirely dependent on the assessment of prognosis, which will only become clearer with the benefit of time. If Annie had signs of more severe neurological injury or recurrent medical issues like aspiration events or high caregiving burdens at present, then the balance might be skewed away from benefit.

The American Academy of Pediatrics (AAP) [4] also published clinical guidance on forgoing medically provided nutrition and hydration in children. Similar to the RCPCH guidelines, it states that a balance of relative burdens and benefits must occur within the context of other medical decisions and the goals of care. It outlines situations in which medically provided fluids and nutrition may be morally optional if it does not provide a net benefit. These include situations such as severe congenital or acquired central nervous system (CNS) injuries where children may never possess consciousness or children in a persistent vegetative state. This differs from children in a “minimally conscious state”, where they have reproducible ability to respond to some environmental stimuli and have intermittent awareness of themselves and their surroundings. As it is difficult or impossible to understand these individuals’ subjective experiences, the decision to withdraw or withhold medical interventions must be made carefully, such that “judgements are not inappropriately influenced by prejudice regarding disability”. The report states that “disability alone is not a sufficient reason to forgo medically provided fluids and nutrition”, and the decision should be made on the basis that the benefits outweigh the burdens of the intervention, like all other medical decisions. Clearly, in deciding in Annie’s case, more communication and time may be needed to understand the child’s medical diagnosis and prognosis from all stakeholders’ perspectives, as well as their values, wishes and acceptable level of disability.

### 3.4. The Idea of Uncertainty

**Figure 2:**
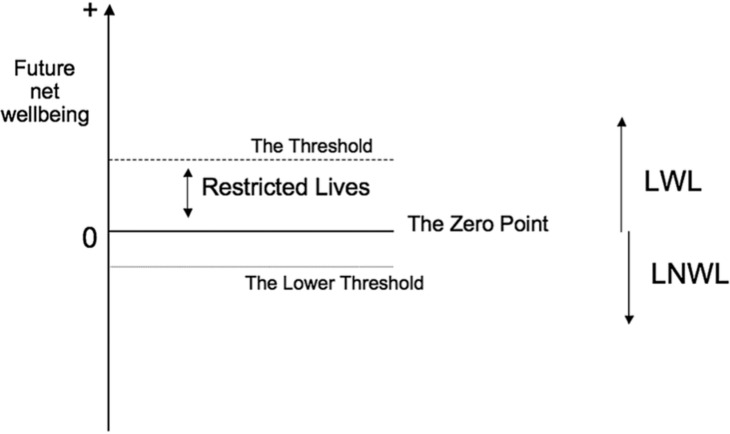
The permissibility of treatment withdrawal from newborn infants based upon predicted future well-being.

Another framework proposed by Wilkinson DJ suggests that there are three possibilities for every newborn—*a life worth living (LWL)*, where future benefits outweigh burdens; *a life not worth living (LNWL),* where future burdens outweigh benefits; and *the zero point*, a life in which future benefits are equal to the burdens (see Figure 2) [16] (p. 22). In contrast to the previous two guidelines, this takes into account the future prognosis of the child. Wilkinson proposes the use of the *Threshold View* wherein life support may be permissibly withdrawn if the infant’s quality of life is below a threshold just above the “zero point”. This view takes into account both prognostic and moral uncertainty, as well as the burden of care, and makes it permissible to withdraw life support from infants who will probably have lives above the zero point. He claims that it “may be worse to allow an infant to live with an LNWL, than to allow a newborn to die who would have had a restricted life”. That said, the clinical presentation of neurological injury in infants is ultimately heterogeneous, with a significant range of variability in symptoms, severity and outcomes. Infants have a greater degree of plasticity and developmental immaturity, and it often takes a long period of time before the extent of impairment is known. This adds to the difficulty in clinical assessment and prognosis-setting when the decision needs to be made for the withdrawal of nutrition and hydration. Hence, some might argue that the greater the uncertainty of the outcome is for newborn infants, the less parental discretion should be given [17]. As in the case of Annie, perhaps more time and expert opinions should be sought to give more clarity on the certainty of her future prognosis so that all stakeholders will be able to come to an amicable agreement on the way forward that will truly be in her best interest. However, if there remains an impasse, given the irreversible nature of the decision-making, the medical team must hold some responsibility and cannot defer to the parents to be the primary determinants of the decision to withhold hydration and nutrition.

## 4. Conclusions

Medical decisions involving the cessation of medical therapies that lead to death are often emotional and difficult. Pervasive uncertainty adds to the complexity involved.

With respect to baby Annie, we propose the following before feeding withdrawal:

1. Understand what suffering and quality of life mean to parents (and providers) and what level of disability is acceptable. This elucidates any underlying difference in value systems among stakeholders that indirectly fosters alliance.
2. Among professional disciplines, examine deeply and pragmatically how long current uncertainty would persist. This provides a time frame before any final decision is made.
3. A ‘safe and close’ community of practice should be set up to address possible moral distress. The ethics committee and peer support are good places to start.

The child’s well-being is vested in a dynamic interplay of sociocultural, ethical and legal factors. A nuanced and customized approach to care that respects the child and family’s unique circumstances and needs is key while guided and supported by healthcare professionals throughout.

## Data Availability

The original contributions presented in the study are included in the article, further inquiries can be directed to the corresponding author.

## Abbreviations

CPR	cardiopulmonary resuscitation
NICU	neonatal intensive care unit
MCDA	monochorionic diamniotic
CPAP	continuous positive airway pressure
EEG	electroencephalogram
RCPCH	Royal College of Paediatrics and Child Health
AAP	American Academy of Pediatrics
CNS	central nervous system
LWL	a life worth living
LNWL	a life not worth living
